# The *melanized* ( *mel* ) locus of *Drosophila melanogaster* is a mutation in CG17065, an N-acetylglucosamine-6-phosphate deacetylase

**DOI:** 10.17912/micropub.biology.002178

**Published:** 2026-05-28

**Authors:** Maya Chandar, Emily Harclerode, Elizabeth Kim, Jasmine J. S. Marsh, Adolya Moore, William E. Moragne III, Danira R. Mukhamedyarova, Ishaan Narsinghani, Cody J. Powell, RyAnn Pryor, Radhika Subramani, Gregory M. Blazek, Mikaela Inessa Chandra, Arjun Ramesh, Stephanie M. Fogerson, Tania Guerrero-Altamirano, Eric P Spana

**Affiliations:** 1 Department of Biology, Duke University, Durham, NC, US

## Abstract

Mutations in the *melanized (mel)* gene of *Drosophila melanogaster* display darker body pigmentation and an enhanced trident pattern on the notum. By complementation, we localized the gene underlying *mel* to *CG17065* , which encodes an N-acetylglucosamine-6-phosphate deacetylase. We find that *mel ^1^* is a missense mutation in a ligand-binding amino acid and that *mel ^1^* fails to complement a protein trap allele of CG17065. Using this allele, we find *mel* /CG17065 is ubiquitously expressed across larval and ovariole tissues. N-acetylglucosamine-6-phosphate deacetylase is predicted to act as an antagonist to the production of UDP-GlcNAc, the precursor for chitin biosynthesis and O- and N-linked glycosylation.

**Figure 1. D. melanogaster melanized ( mel ) maps to CG17065 and is ubiquitously expressed:** **(A)** Wild type Oregon-R (OreR) *D. melanogaster* female shows normal body pigmentation. ( **B)** A female of the genotype *mel ^1^ mal ^1^* shows a slightly darker body and pronounced trident pattern due to *mel ^1^* and dull-colored eyes due to *mal ^1^* . **(C)** A female of the genotype *mel ^1^ mal ^1^* /Df(1)BSC644 shows the same pigmentation as *mel ^1^ mal ^1^* homozygotes. **(D)** A female of the genotype *mel ^1^ mal ^1^* / CG17065 ^CR^ has the same pigmentation phenotype as **B** and **C** . **(E-H)** Decapitated heads of the same genotypes as **A-D** shows the hypermelanized region in the ocellar region and near the interorbital bristles (especially **F** & **H** ). **(I)** An approximately 75kb region of the X chromosome shows the overlap of the two duplications (light blue) that rescue *mel ^1^* . Dp(1;3)DC374 extends off the map on the right, and Dp(1:3)DC554 extends off the map to the left. Five protein coding genes: Rab10, CG42577, Inx6, & CG1504 in dark blue and CG17065 in purple, and two non-coding RNA genes (CR45504, CR45505 in orange) lie within this region. Arrowheads indicate the four CR alleles used for complementation. **(J)** The *melanized* /CG17065 gene consists of three splice isoforms. The *mel ^1^* mutation is shown in red. The CG17065CR allele would terminate translation of the gene after exon 1. The CG17065 ^EY^ insertion is found in the 5’UTR of all three isoforms. **(K)** A close-up view of the binding site of the human ortholog of *mel* /CG17065 (AMDHD2) bound to N-glucosamine-6-phosphate (green outline). The histidine sidechain (red outline) makes a hydrogen bond to the C-1 hydroxyl group of the glucosamine ring. This histidine is mutated to leucine in *mel ^1^* . **(L-M)** Expression of *mel* /CG17065 as visualized by the GAL4 found in the CG17065 ^CR^ insertion. Red is the expression of UAS-mCherry-NLS, blue is DAPI stained nuclei, and green is phalloidin. The merge of all three colors is shown in the fourth column. Scale bar is 100 μm. **(L)** mCherry-NLS is expressed in all the nuclei of the larval salivary gland (arrowhead) and associated fat body (arrow) at 200X. **(M)** mCherry-NLS is expressed in all nuclei of the larval midgut at 200X. **(N)** mCherry-NLS is expressed in both cell types of the larval Malpighian Tubule: the Stellate cells (arrow) and Principal cells (arrowhead) at 400X. **(O)** mCherry-NLS is expressed in all follicle cells in this Stage 9 egg chamber at 200X.

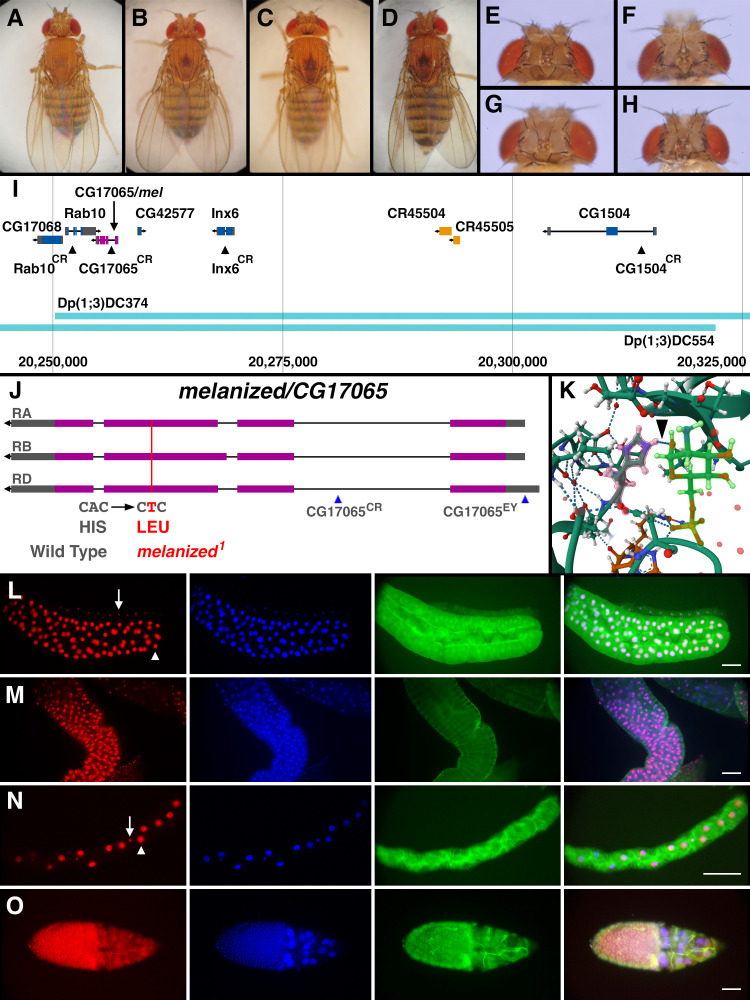



## Description

The body pigmentation of the adult *Drosophila melanogaster* is produced through a combination of melanin and two sclerotization pathways, all of which are derived from dopamine (Wright, 1987). Dopamine is synthesized from tyrosine via Tyrosine Hydroxylase (encoded by the *pale* gene) and Dopa Decarboxylase (encoded by the *Ddc* gene) (reviewed in Yamamoto & Seto, 2014). That dopamine pool can be used by several pathways: it can be converted to melanin via the genes *yellow* , *yellow-f* , *straw* , and others (Han et al., 2002; Riedel et al., 2011; Sickmann et al., 2017); it can be converted to N-acetyldopamine (via the *speck* gene) for incorporation into NADA sclerotin (Spana et al., 2020), or it can be combined with beta-alanine (by the *ebony* gene) to make NBAD sclerotin (Richardt et al., 2003). A recent RNAi screen identified over 100 genes autonomously required for adult pigmentation (Deal et al., 2026). However, because of its rich research history, there are still viable strains of *D. melanogaster* available with body color defects that are unannotated or recently de-orphaned (Dean et al., 2022). Here we map the melanized ( *mel* ) gene via genetic complementation, and document its phenotype and expression.

Alleles of the *mel* gene have been isolated twice ( *mel ^1^* and *mel ^2^* ) (Fahmy, O.G., Fahmy, M., 1958), however *mel ^2^* has been lost. *mel ^1^* has been described as having a darker body color and an especially prominent ‘trident’ on the thorax as well as dull red eyes and occasional upturned wings. It has been mapped genetically to 1-64.1 (Fahmy, O.G., Fahmy, M., 1958) and cytologically to 19B-C (Chovnick et al., 1969). Of the three phenotypes associated with *mel* , we could only use complementation tests by scoring the body color. The wing phenotype was not penetrant enough and we could not distinguish the eye color phenotype (and the *mel ^1^* chromosome also carries an eye color mutation, *maroon-like* , *mal ^1^* ).

We first confirmed the phenotype of the *mel ^1^* allele. As expected, when compared to wild type Oregon-R (OreR) ( **Figure 1A** ) *mel ^1^* had a dark trident and darker body color ( **Figure 1B** ). Additionally, the *melanized* phenotype was prominently displayed in the ocular ridge around the eyes ( **Figure 1E, F** ). Next, to map *mel ^1^* , we performed a series of complementation tests using deficiency and duplication stocks (abbreviated Df and Dp, respectively) to localize the mutation. We found that *mel* failed to complement Df(1)HM44 exactly as described in 1986 (Miklos et al., 1986) and crosses with wild type Oregon-R showed no dominant effect of *mel* , no thoracic tridents or darker bodies. We then tested five molecularly defined deficiency strains: Df(1)ED7635, Df(1)BSC644, Df(1)BSC872, Df(1)BSC648, and Df(1)BSC586 that span from 19A1 to 19E7. Of these, only Df(1)ED7635 complemented *mel* . That places *mel* between the left breakpoint of Df(1)BSC644 and the right breakpoint of Df(1)BSC872: the 19C1-4 range. When in trans to *mel ^1^* , Df(1)BSC644 fails to complement and shows the same thoracic *mel* phenotype as *mel ^1^* homozygotes, indicating that *mel ^1^* is likely a strong, if not null, allele ( **Figure 1C** ). However, *mel ^1^* in trans to a deficiency (either Df(1)BSC644 or Df(1)BSC872) showed a less severe hyperpigmentation near the interorbital bristles ( **Figure 1G** ). We did not score heads of the other non-complementing deficiencies, and it is not clear how these two deficiencies that overlap by ~87 kb/9 protein coding genes might fail to complement *mel* in the thorax, but give a less severe head phenotype. To further refine this mapping, we found that, when crossed with *mel ^1^* , Dp(1;3)DC374 and Dp(1;3)DC554 rescue the wild-type pigmentation in both the thorax and head, while Dp(1;3)DC553; Dp(1;3)DC556; and Dp(1;3)DC409 do not. Taken together, these complementation results localized the *mel* locus to the ~72 kb region between positions 20,250,237–20,322,155, which contains five candidate protein coding genes: Inx6, Rab10, CG1504, CG42577, and CG17065 ( **Figure 1I** ). Mutations in four of these genes were individually tested by complementation with *mel ^1^* . Only an allele of CG17065, CG17065 ^CR^ , failed to complement *mel¹* ( **Figure 1D, H** ). Further, flies homozygous for the CG17065 ^CR^ mutation had the same dark body and trident phenotype as *mel ^1^* . These data gave us a strong inclination that *mel ^1^* was a mutation in the CG17065 gene, and to test this, we amplified and sequenced CG17065 from wild-type OreR and *mel ^1^* flies. We found a single base change that causes a mis-sense mutation: histidine to leucine at amino acid 283 ( **Figure 1J** ). A second insertion in CG17065 (CG17065 ^EY^ ) was found to complement both *mel ^1^* and CG17065 ^CR^ . The CG17065 ^EY^ insertion is located in the 5’ UTR of all three splice isoforms; however, it does have a basal promoter and UAS sequences that would facilitate transcription of the CG17065 gene. Attempted mis/overexpression of CG17065 by crossing CG17065 ^EY^ to different GAL4 drivers showed no pigmentation phenotypes or viability issues. Because CG17065 ^CR^ has a *melanized* phenotype and fails to complement *mel ^1^* , and *mel ^1^* has a missense mutation in CG17065, we conclude that CG17065 is the *melanized* gene. CG17065 encodes N-acetylglucosamine-6-phosphate deacetylase (abbreviated hereafter as GlcNAc-6-P deacetylase).

To understand how a single amino-acid substitution could generate such a pronounced loss of function, we examined the position of the H283L mutation within the GlcNAc-6-P deacetylase structure. We used the model for the human ortholog of GlcNAc-6-P deacetylase (called AMDHD2) because it had a structure available that included the bound ligand ( https://doi.org/10.2210/pdb7NUT/pdb ) (Kroef et al., 2022). From this, we found that Histidine 283 (Histidine 272 in human ortholog) lies in close proximity to the bound substrate, N-acetylglucosamine-6-phosphate ( **Figure 1K** ). In the wild-type protein, the imidazole side chain of Histidine 283 is positioned to form a stabilizing hydrogen bond with the O atom of the C1-hydroxyl group of the ligand, likely contributing to the proper orientation and affinity of substrate binding. Substituting histidine with leucine eliminates this polar, hydrogen-bonding-capable residue and introduces a hydrophobic side chain into an otherwise polar microenvironment. This change would then disrupt favorable electrostatic and hydrogen-bonding interactions and alter the geometry of the ligand-binding pocket, thereby reducing substrate affinity and reducing GlcNAc-6-P deacetylase activity.

To assess GlcNAc-6-P deacetylase expression across tissue types, we utilized the CG17065 ^CR^ allele. This CRISPR insertion is a CRIMIC insertion that traps and truncates the *mel* /CG17065 transcript and initiates expression of GAL4 from *mel* /CG17065 mRNA (Kanca et al., 2022). Crossing this GAL4 line to a UAS-mCherry-NLS will reproduce the *mel* /CG17065 expression pattern. A colocalization approach involving Phalloidin and DAPI fluorescent staining was additionally used to visualize F-actin and nuclei staining, respectively. Here, we see near ubiquitous expression across *Drosophila* tissues. In third instar larvae, we see expression in imaginal discs (wing, eye-antennal, leg), brain, trachea, and body wall. Here we document notable expression in the salivary gland nuclei (arrowhead in Figure 1L ) as well as associated fat body cells (arrow); the midgut ( Figure 1M ), and both cell types in Malpighian tubules: Stellate cells (arrow) and Principal cells (arrowhead in Figure 1N ). We also examined ovarioles in adult females and see expression in ovarian follicle cells as seen in this stage 9 egg chamber ( Figure 1O ). We chose these tissues to show because they have large, clearly resolvable nuclei that can be recorded clearly by our lab’s microscopy equipment, not because of any potential relation to the phenotype. Because GlcNAc-6-P deacetylase is expressed in every tissue, it could be acting either autonomously or non-autonomously to produce the *melanized* phenotype.

How might a loss of function in GlcNAc-6-P deacetylase cause the pigmentation phenotype found in *melanized* ? It is known that GlcNAc-6-P deacetylase, an enzyme involved in the hexosamine biosynthesis pathway, is responsible for the deacetylation of N-acetylglucosamine-6-phosphate (GlcNAc‑6‑P) to glucosamine-6-phosphate (Glc-6-P) (Paneque et al., 2023). GlcNAc-6-P is two enzymatic steps from UDP-GlcNAc, which is the starting material for both O- and N-linked glycosylation, as well as chitin formation. It is possible that the loss of GlcNAc-6-P deacetylase might cause an excess of GlcNAc-6-P, and thus an excess UDP-GlcNAc. Because UDP-GlcNAc is such a keystone molecule in multiple pathways, there is no shortage of potential mechanisms: Tyrosine Hydroxylase is regulated by O-linked glycosylation (da Costa Rodrigues et al., 2025); in *Drosophila* , loss of the *hippo* signaling pathway causes excess cuticle pigmentation (Gibson et al., 2025), and the kinase in the middle of the pathway ( *warts* ) has a human ortholog, LATS1 (Large Tumor Suppressor Kinase 1) that is regulated by O-linked glycosylation (Meng et al., 2026); and a different enzyme in the hexosamine biosynthesis pathway is required for mouse and human hair pigmentation (Tiede et al., 2021). Future experiments with *melanized* in cuticle pigmentation (such as mosaics to determine autonomy), the protein glycosylation pathways (such as looking for genetic interactions with mutations in the N- or O-linked pathways), and the chitin biosynthesis pathways (testing chitin composition, for example) should provide answers for how loss of the GlcNAc-6-P deacetylase can cause the *melanized* phenotype.

## Methods

**Fly Imaging:** To image adult flies, 5-10 day old females were anesthetized using FlyNap (Carolina Biological Supply Company) by placing a saturated FlyNap swab in the food vial for 2-5 minutes which allowed immobilization and was verified by tapping. Excess product was removed to ensure survival. Once anesthetized, flies were transferred to a white surface for optimal visibility and oriented with their dorsal side up. A Leica MZ12.5 stereomicroscope equipped with a Nikon D300S camera was adjusted to 50x magnification, and the camera was focused on the notum while keeping wings and legs in view. A hollow printer-paper cylinder (~2.5 cm diameter, ~2.5 cm high) was placed around the specimen to reduce ring-light reflection on the thorax while maintaining clear visualization of the fly in its natural position. This protocol is based on some of the methods seen here: http://gompel.org/wp-content/uploads/2015/09/Depth_of_field_Drosophila-photography.pdf

To image heads, females were anesthetized with CO₂. The heads were removed from the bodies using a razor blade and positioned on the end of a wooden toothpick (GoodCook Everyday Shake-A-Pick toothpick) and adhered with L.A. Colors clear topcoat nail polish. Once secure, the toothpick was positioned on a ~1 cm ^3^ piece of modeling clay so that the dorsal side of the head was up. The total magnification was set to 63x and focused on the orbital bristle region to visualize the hyperpigmented patches. The heads were photographed above a BEHR paint swatch (Bluebird PPU15-12M, Home Depot) for optimal visibility.

**Genomic DNA isolation from Drosophila:** Genomic DNA was isolated from OreR, *mel ^1^ mal ^1^* , and *w ^1118^ CG17065 ^CR^* flies using Qiagen’s DNeasy Blood and Tissue kit (Cat no. / ID. 69504) and stored at -20ºC after isolation. For amplifying the CG17065EY region we used a squishing buffer prep (Gloor, 1992).

**PCR & Sequencing:** We used the genome sequence (r6.65) available on FlyBase and Primer3Plus ( www.primer3plus.com ) to design primers. The CG17065.CP-F and CG17065.CP-R primers were used to amplify the entire CG17065 locus using these conditions: 93° for 3 minutes to activate the polymerase, (93° for 30 seconds, 55° for 15 seconds, 72° for 2.5 minutes) x 35 cycles and a final 10-minute extension at 72°. For the CG17065 ^CR^ insertion, we increased the extension time to 5 minutes. To verify the EY insertion, we used combinations of the primers: CG17065.EY-F and CG17065.EY-R (genomic primers) and the P-end primer (which is to the P-element inverted terminal repeat and will extend off both ends) under these conditions: 93° for 3 minutes to activate the polymerase, (93° for 30 seconds, 55° for 15 seconds, 72° for 0.5 minutes) x 35 cycles and a final 10-minute extension at 72°. We performed PCR using the UltraRun LongRange PCR Kit (QIAGEN). PCR products were sequenced using Oxford Nanopore technology by Plasmidsaurus.

**Larvae/Ovariole staining:** Wandering third instar larvae were dissected under PBS by tearing at the halfway point, and then inverting both the anterior and posterior ends. Mature female ovarioles were dissected by placing the female under PBS and removing the posterior end with ovarioles still attached. Dissected tissue was fixed for 30 minutes in PBS+4% paraformaldehyde. After fixation, the tissue was briefly rinsed 3 times with PBS+0.1% Triton X-100 (PBS+T), and then washed for 30 minutes in the same. Tissue was then stained by incubation with 1 µg/ml DAPI and 1:500 Alexa Fluor 488-Phalloidin for 30 minutes. After 3 brief washes in PBS+T, tissue was mounted in Vectashield+DAPI.

## Reagents

**Table:** 

**Reagent Type**	**Designation**	**Identifier**	**Reference/Source**	**Additional Information**
Genetic reagent (D. *melanogaster* )	OreR	N/A	Lab strain	Genotype: OreR
Genetic reagent (D. *melanogaster* )	mel ^1^ mal ^1^	Derived from BDSC 3973	Bloomington Drosophila Stock Center	Genotype: mel ^1^ mal ^1^ Derived from *y ^1^ v ^1^ mel ^1^ mal ^1^* by recombination with OreR
Genetic reagent (D. *melanogaster* )	CG17065 ^CR^	Derived from BDSC 93959	Bloomington Drosophila Stock Center	Genotype: *w ^1118^* CG17065 ^CR60133-TG4.1^ Derived from y* w* CG17065 ^CR60133-TG4.1^ by recombination with *w ^1118^*
Genetic reagent (D. *melanogaster* )	Rab10 ^CR^	BDSC 605429	Bloomington Drosophila Stock Center	y ^1^ w* TI{CRIMIC.TG4.1}Rab10 ^CR71289-TG4.1^
Genetic reagent (D. *melanogaster* )	Inx6 ^CR^	BDSC 600096	Bloomington Drosophila Stock Center	y ^1^ w* TI{KozakGAL4}Inx6 ^CR70789-KO-kG4^
Genetic reagent (D. *melanogaster* )	CG1504 ^CR^	BDSC 93929	Bloomington Drosophila Stock Center	y ^1^ w* TI{CRIMIC.TG4.0}CG1504 ^CR02588-TG4.0^
Genetic reagent (D. *melanogaster* )	CG17065 ^EY^	BDSC 16381	Bloomington Drosophila Stock Center	* y ^1^ w ^67c23^ P{EPgy2}CG17065 ^EY06065^*
Genetic reagent (D. *melanogaster* )	Dp(1;3)DC374	BDSC 32282	Bloomington Drosophila Stock Center	Genotype: *w ^1118^* ; Dp(1;3)DC374
Genetic reagent (D. *melanogaster* )	Dp(1;3)DC554	BDSC 33505	Bloomington Drosophila Stock Center	Genotype: *w ^1118^* ; Dp(1;3)DC554/TM6C, Sb ^1^
Genetic reagent (D. *melanogaster* )	Dp(1;3)DC556	BDSC 33506	Bloomington Drosophila Stock Center	Genotype: *w ^1118^* ; Dp(1;3)DC556/TM6C, Sb ^1^
Genetic reagent (D. *melanogaster* )	Dp(1:3)DC371	BDSC 32281	Bloomington Drosophila Stock Center	* w ^1118^* ; Dp(1;3)DC371
Genetic reagent (D. *melanogaster* )	Dp(1;3)DC553	BDSC 33504	Bloomington Drosophila Stock Center	* w ^1118^* ; Dp(1;3)DC553/TM6C, Sb1
Genetic reagent (D. *melanogaster* )	Dp(1;3)DC409	BDSC 30799	Bloomington Drosophila Stock Center	* w ^1118^* ; Dp(1;3)DC409
Genetic reagent (D. *melanogaster* )	Df(1)HM44	BDSC 6279	Bloomington Drosophila Stock Center	Genotype: Df(1)HM44, *y ^1^ w ^a^* / Dp(1;Y)y ^+^ mal ^+^
Genetic reagent (D. *melanogaster* )	Df(1)BSC644	BDSC 25734	Bloomington Drosophila Stock Center	Genotype: Df(1)BSC644 *w ^1118^* / Binsinscy
Genetic reagent (D. *melanogaster* )	Df(1)BSC872	BDSC 29995	Bloomington Drosophila Stock Center	Df(1)BSC872, *w ^1118^* BSC872/FM7h/Dp(2;Y)G, P{hs-hid}Y
Genetic reagent (D. *melanogaster* )	Df(1)BSC586	BDSC 25420	Bloomington Drosophila Stock Center	Df(1)BSC586, *w ^1118^* /Binsinscy
Genetic reagent (D. *melanogaster* )	Df(1)BSC648	BDSC 25738	Bloomington Drosophila Stock Center	Df(1)BSC648, *w ^1118^* /FM7h/Dp(2;Y)G, P{ *w ^+mC^* =hs-hid}Y
Genetic reagent (D. *melanogaster* )	UAS-mCherry.nls	BDSC 38424	Bloomington Drosophila Stock Center	w*; P{w ^+^ , UAS-mCherry.NLS}3
Chemical	FlyNap		Carolina Biological Supply Company	
Chemical	Vectashield + DAPI	H-1200	Vector Laboratories, Burlingame	
Chemical	Alexa Fluor 488-Phalloidin	A12379	Invitrogen	
Software	Protein Data Bank	N/A	https://www.rcsb.org/	
PCR Kit	UltraRun LongRange PCR Kit	Cat no. / ID. 206442	QIAGEN	
Oligonucleotide	CG17065.CP-F primer	TAGCATTCGCATTTGCATTCG	Eton Bio	
Oligonucleotide	CG17065.CP-R primer	GCACTGGAATTGGTTTCTCAGT	Eton Bio	
Oligonucleotide	CG17065.EY-R primer	GTCGGGGGTGGCGCAAGG	Eton Bio	
Oligonucleotide	CG17065.EY-F primer	TCTGGCTCCTCGCTGTTG	Eton Bio	
Oligonucleotide	P-end primer	CGACGGGACCACCTTATGTTATTTCATCATG	Eton Bio	
